# “The storm has arrived”: the impact of SARS-CoV-2 on medical students

**DOI:** 10.1007/s40037-020-00592-2

**Published:** 2020-05-26

**Authors:** Jennifer M. Klasen, Akschaya Vithyapathy, Bjoern Zante, Sarah Burm

**Affiliations:** 1 Clarunis, Department of Visceral Surgery, University Center for Gastrointestinal and Liver Diseases, University Hospital Basel, Basel, Switzerland; 2 Medical University of Vienna, Vienna, Austria; 3 Department of Intensive Care Medicine, Inselspital, Bern University Hospital, University of Bern, Bern, Switzerland; 4 Division of Medical Education, Dalhousie University, Halifax, Nova Scotia Canada

**Keywords:** SARS-CoV‑2, COVID-19, Coronavirus, SARS, Medical students, Medical Education, Pandemic

## Abstract

In a few weeks, the global community has witnessed, and for some of us experienced first-hand, the human costs of the COVID-19 pandemic. There is incredible variability in how countries are choosing to thwart the disease’s outbreak, sparking intense discussions around what it means to teach and learn in the era of COVID-19, and more specifically, the role medical students play in the midst of the pandemic. A multi-national and multi-institutional group made up of a dedicated medical student from Austria, passionate clinicians and educators from Switzerland, and a PhD scientist involved in Medical Education from Canada, have assembled to summarize the ingenious ways medical students around the world are contributing to emergency efforts. They argue that such efforts change COVID-19 from a “disruption” to medical students learning to something more tangible, more important, allowing students to become stakeholders in the expansion and delivery of healthcare.

As rapid escalation of COVID-19 continues, the world has taken extreme measures to contain the virus. As of mid-March, Western Europe is in complete lockdown. Italy has been in state of emergency [1, 2], as our healthcare colleagues continue fighting against SARS-CoV‑2 and for their patients. France and Spain have imposed a nationwide curfew [3, 4]. Around the globe, the last few weeks have been spent preparing for and anticipating the inexorable surge of moderately to seriously ill patients suffering from the virus. We are trying to learn from the experiences of our Italian neighbors and our Asian colleagues. The pressure on our healthcare system is building. The world feels irrevocably altered.

Amid our central concerns for the care of our patients and the safety of our healthcare providers, another question emerges: What is the role of medical students during this pandemic? For many, their exposure to the clinical environment has come to a grinding halt. The pace at which this outbreak has spread leaves many medical students sidelined, wondering what role they can play to meaningfully contribute [5].

As the crisis drags on, a larger, more perplexing question emerges: What is the *clinical role* of medical students during this pandemic? Responses suggest that, as a global community, medical education has a varied and fluid stance on this question [6]. Take the situation in Switzerland, where educators and students are in regular discussion about the role senior students play in this unique situation. In some cases, that discussion is shaped by national factors. For instance, some students have been ordered back to their home countries, such as Germany, where their help might be needed in the coming days. Other factors are more personal, as exemplified by a recent incident in which our local hospital authorities encouraged a medical student in the clerkship year to go home to take care of a sick child. The student expressed a desire to “stay and help where I can … avoid infected patients, write notes or something”. In this situation, the individual faced the moral dilemma of having to choose between caring for a loved one and fulfilling their professional obligations. Other medical clerks were allowed to stay, with variable engagement in clinical activities and equipped with appropriate protective equipment. Our point is not to say that sending the student home was the right or wrong decision; rather, that such decisions these days are not uncommon and should be made conscientiously because they involve fundamental issues of equity and ethics.

Worldwide, healthcare systems and medical education authorities have responded variably to medical students’ participation in the COVID-19 healthcare crisis. Some hospitals have banned students from involvement in any clinical activities, while others have been more targeted, keeping students away from wards where COVID-19 patients are treated, or keeping them out of emergency departments and ICUs [7, 8]. Still other hospitals have allowed students to stay and work. The precariousness of this global emergency leaves medical students confronting ethical and practical dilemmas never previously considered [5]. In some of these situations, students may have little choice, particularly those from single or low income households: either they go to work or they stay at home with no salary, no social insurance and no certificate for their medical studies resulting in postponement of their medical graduation [9].

Other countries have considered this moment of crisis as an opportunity to introduce remote medical training [10] and explore how medical students can apply their growing medical knowledge in real-life healthcare contexts and situations. For example, public health officials across Canada have begun recruiting senior medical students to assist in the necessary task of tracking down individuals who have been in contact with infected patients [11]. The University Hospital of Basel, Switzerland, has trained and mobilized hundreds of volunteer medical students to assist frontline workers in massive testing, supporting primary care providers and overwhelmed emergency departments in triaging patients. Other countries, such as the United States, the United Kingdom, Italy, and Ireland have fast-tracked final year medical students, hoping that the influx of new physician graduates will mitigate the potential catastrophic consequences the healthcare system faces [12–16]. As countries struggle to reinforce their health workforce, this may be viewed as a more ethical strategy and practical solution than the alternative of calling back retired physicians, who are at statistically higher risk of becoming seriously ill from COVID-19 [17].

Seeing this variety in responses, one has to conclude that we did not consolidate our learning from the SARS “storms” we have seen in the past, although in 2004 medical educators were recommending to do so [18].

Here we are in 2020, in the eye of another storm, faced with the question of of how we should approach the role of (senior) medical students in a pandemic. Sending students home makes sense on a number of fronts [19–21]. A worsening shortage of protective equipment means this scarce resource should be reserved for healthcare providers, not used by students doing clinical placements [22, 23]. The surge of clinical care requires that faculty must devote themselves to saving lives, rather than teaching novices. At the same time, most students in their final clinical year are going to be doctors in the coming few months, meaning that they have the knowledge to assist in some capacity. Questions about health insurance for students also impact the decision [24]. Finally, we must consider emerging evidence that medical students, mostly members in the 20–29 year age group who may be silent vectors of the virus, may introduce another danger to the healthcare system [25].

Looking to history, we would draw mixed conclusions about whether we should send students home or engage them as a resource for sustaining our healthcare system in the pandemic’s coming surge. On the one hand, during the “Spanish flu” 1918/19, medical and nurse students were explicitly used to support the understaffed healthcare system [26]. On the other hand, during the SARS epidemic 2003, Hong Kong and Toronto reported that students became infected because they approached index patients, not knowing about their infection and not using protection [27, 28]. Deployed without appropriate protection and education, students are both a less expensive healthcare workforce, and a more vulnerable one [9].

Prioritizing the safety and wellness of medical students is of paramount concern. However, let’s not dismiss the impromptu learning opportunities this pandemic presents. Working on the frontlines boosts critical thinking skills and enables medical students to engage in real-world advocacy work. Furthermore, they can witness and learn from the crucial discussions unfolding related to prioritization of care and resource management, such as the rationing of protective equipment and ventilators, and the moral and ethical implications informing these key decisions.

It is important to recognize that medical students are not responding passively in the debate around their involvement in the COVID-19 pandemic. We have seen evidence from around the world that many are passionate in their wish to contribute during this time of crisis [29]. Many students are volunteering their time, both in clinical settings and in community roles. They are eager to help patients and support their future colleagues, not only to comply with the ethics of the profession but also to meet their desire for civilian service in society. For example, German students have launched a website that offers frontline healthcare providers immediate access to a network of people seeking and offering help during the current COVID-19 situation [30]. Hospitals, primary care givers and public health departments across Germany, the Netherlands, Austria and Switzerland are using the website (match4healthcare.de) as an online platform to inform student volunteers about their needs. Their Facebook group gained more than 12,000 members in the first few days. Similarly, in Canada, medical student groups have organized to provide support for beleaguered healthcare staff in their institutions, offering to help with childcare, errands, grocery delivery and other household tasks in ways that observe social distancing policies [31, 32].

What, then, should we do? Students desperately want to help and, as the surge of cases continues to rise, we are going to desperately need them. But we must have meticulous, ethical procedures in place as we engage them as novice members of the clinical workplace. We must balance the clinical and educational value of direct patient contact with the risk of exposure to the virus. In such a crisis, no one should be pressured to work. Medical students should have a solid work contract and be covered by proper health insurance by the hospital. And their learning needs during this time must be addressed to keep them safe. If medical students choose to join the frontlines, training and instructions in the corresponding tasks are required. Yes, they will work outside their sphere of competence, just as many of us will be working outside of ours too. But supervision, feedback, and evaluation of students must not be abandoned, in spite of tight faculty resources. We need to assess which tasks students might safely do independently with appropriate training and protective procedures, and which they may be able to do with some degree of expert supervision [33]. The balance of clinical and educational priorities will certainly shift in this time of urgent clinical need, but both must receive attention if we are to involve medical students in an ethically responsible manner. The healthcare system will be stretched to the margins and probably beyond. It is to be hoped that medical students will not become victims of the circumstances but rather receive the attention they deserve. Preparing them for expedited entry into the clinical workforce will be of immediate benefit to their communities and will benefit the global medical education community long after the coronavirus has run its course.

With the support of dedicated medical students, and from reviewing current literature and a number of electronic sources [34–37], we have developed the following recommendations:

Recommendations:

1. Students must know and recognize the symptoms of the disease as well as how to protect patients and themselves.
2. The assignment of students can be rescheduled at any time if there is an increase in knowledge or a redistribution of resources; there should be a constant re-evaluation by the supervising authority.
3. An introduction to the corresponding tasks must not be neglected; briefing and debriefing activities should take place regularly.
4. We suggest creating and maintaining the best possible conditions if we seek to deploy medical students during such a crisis while mentoring them.
5. We do not know exactly what and how students learn in such crisis situations and what coping strategies they have, so we as educators have to be aware of the need to support them even more than usual during such uncertain times. Prioritizing medical students’ safety and wellbeing is essential.
